# Systematic Investigation of Scutellariae Barbatae Herba for Treating Hepatocellular Carcinoma Based on Network Pharmacology

**DOI:** 10.1155/2018/4365739

**Published:** 2018-11-21

**Authors:** Benjiao Gong, Yanlei Kao, Chenglin Zhang, Fudong Sun, Huishan Zhao

**Affiliations:** ^1^Affiliated Yantai Yuhuangding Hospital of Qingdao University, Yantai, China; ^2^Yantai Hospital of Traditional Chinese Medicine, Yantai, China

## Abstract

As the fifth most common type of malignant cancers globally, hepatocellular carcinoma (HCC) is the second leading cause of cancer-related mortality worldwide. As a long-time medicinal herb in Traditional Chinese Medicine (TCM), Scutellariae Barbatae Herba (SBH) has also been used for treating various cancers including HCC, but its underlying mechanisms have not been completely clarified. Presently, an innovative network-pharmacology platform was introduced to systematically elucidate the pharmacological mechanisms of SBH against HCC, adopting active ingredients prescreening, target fishing, and network analysis. The results revealed that SBH appeared to work on HCC probably through regulating 4 molecular functions, 20 biological processes, and hitting on 21 candidate targets involved in 40 pathways. By in-depth analysis of the first-ranked signaling pathway and hit genes, only TTR was highly and specially expressed in the liver tissue. TTR might play a crucial role in neutrophil degranulation pathway during SBH against HCC. Hence, TTR might become a therapeutic target of HCC. The study investigated the anti-hepatoma mechanisms of SBH from a holistic perspective, which provided a theoretical foundation for further experimental research and rational clinical application of SBH.

## 1. Introduction

Hepatocellular carcinoma (HCC) is the fifth most common type of malignant cancer globally and the second leading cause of cancer-related mortality worldwide [1]. The morbidity of HCC is increasing and more than 50% of new cases are diagnosed in China each year [2, 3], while the median survival of patients with advanced HCC is less than 5 months [4, 5]. The high morbidity and mortality were attributed to diagnosis executed at an advanced stage [6]. The five-year survival rate is still less than 30% among patients subjected to hepatectomy [7]. Surgical resection, transarterial chemoembolization (TACE), tumor ablation, and liver transplantation are current treatment modalities and Sorafenib is the only drug approved by FDA [8, 9]. Unfortunately, only TACE and the drug Sorafenib have been shown to provide a survival benefit for patients with advanced HCC (stage II-III) [10, 11].

Scutellariae Barbatae Herba (SBH) is originated from the dried entire plant of Scutellaria Barbata D. Don in the Labiatae family [12], which is natively distributed throughout Korea and southern China [13]. The herb is well renowned in Traditional Chinese Medicine (TCM) as Ban-Zhi-Lian and has been used for hundreds of years in Asian countries. Conducted by TCM theory, SBH possesses effects of heat-clearing, detoxifying, removing blood stasis, and diuretic swelling and has been utilized for treating boils, swollen poison, sore throat, and venomous snake bites for thousands of years in China [14]. Moreover, SBH has also been used for treating primary liver cancer, lung cancer, and carcinoma of uterine cervix, and it is also indicated for more types of cancers in combination with other Chinese herbal medicines [15, 16]. Modern pharmacological studies have illuminated that SBH possesses antioxidant [13], anticancer [17], antiangiogenesis, etc. activities [18]. More importantly, studies demonstrated that SBH presents effect-enhancing and toxicity-reducing actions for chemotherapy in Hepatoma H22 tumor-bearing mice and a protective effect against liver damage induced by various hepatotoxins [16, 19]. Therefore, it is worthwhile to elucidate the potential mechanisms of anti-hepatoma effect.

TCM has the characteristics of multiple components, multiple targets, and synergistic effects [20], which lead to some problems such as unclear mechanisms of action and unclear substance bases. Thereby, it is comparatively difficult to detect the accurate mechanisms of TCM through conventional experimental methods systematically and comprehensively [21]. It is necessary to illuminate scientific bases and potential mechanisms of TCM exploring new approaches. Network pharmacology, first proposed by Andrew L Hopkins, is a systematic analytical way based on the interaction network of diseases, genes, protein targets, and drugs [22], which has substantially promoted the mechanistic studies of herbal medicines [23, 24]. Network pharmacology (sometimes also called systems pharmacology) integrates systems biology, omics, and computational biology to reveal the mechanism of drug action from the overall perspective, which possesses integrity, synergy, and dynamic characteristics [25]. The characteristics coincide with those of the holistic theory of TCM. Thus, we employed network pharmacology to dissect the anti-hepatoma of SBH.

**Figure 1:**
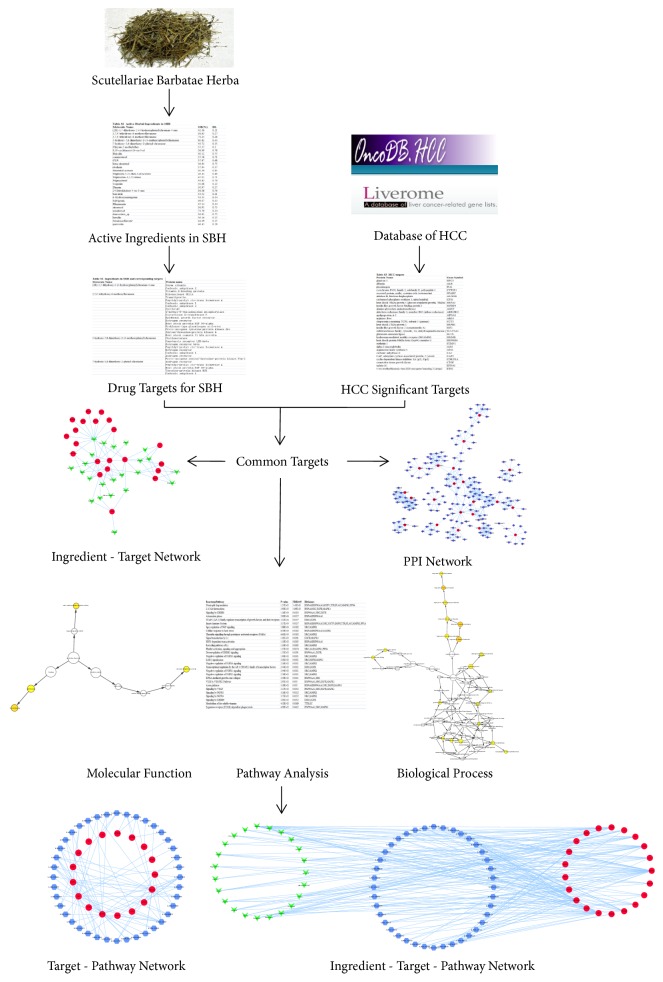
Workflow for SBH against HCC.

In this study, we recruited active ingredients of SBH based on Oral Bioavailability (OB) and Drug-Likeness (DL) and predicted respective targets using pharmacophore mapping approach. HCC significant targets were retrieved from two public databases and mapped to predicted targets of active ingredients for SBH to get common targets. The common targets were executed GO and pathway analysis, which revealed the major biological processes and molecular functions and involved pathways during SBH against HCC. Then, the interaction networks were visualized via Cytoscape software, containing the network between common targets and associated interacting proteins and networks among active ingredients, common targets, and pathways. The workflow of the study on SBH against HCC based on network pharmacology was shown in Figure 1.

## 2. Materials and Methods

### 2.1. Active Ingredients in SBH

The chemical ingredients were obtained from the Traditional Chinese Medicine Systems Pharmacology Database [26] (TCMSP, http://ibts.hkbu.edu.hk/LSP/tcmsp.php), which provides an analysis platform for studying TCM comprehensively. The active ingredients of OB ≥ 30% and DL ≥ 0.18 were selected for subsequent research referring to the most common criteria by TCMSP database. Eventually, 29 active herbal ingredients were selected for SBH (Table S1).

### 2.2. Drug Targets for SBH

The PubChem database [27] (https://pubchem.ncbi.nlm.nih.gov/) is a key chemical information resource, which contains the largest collection of publicly available chemical information. The active ingredients were imported into PubChem database and the 3D molecular structures were exported in the form of SDF files with the exception of 6 compounds without relevant 3D molecular structure information in SBH. The targets cannot be successfully predicted in compounds lacking precise structural information, which were removed. Finally, 23 herbal ingredients with 3D molecular structure information were recruited for further research. PharmMapper Server [28] (http://lilab.ecust.edu.cn/pharmmapper/) is a freely accessed database designed to identify potential target candidates for the given probe small molecules using pharmacophore mapping approach. The predicted drug targets of 23 herbal ingredients were obtained from PharmMapper database. The targets with normalized fit score > 0.9 were harvested as potential targets for SBH after discarding duplicate data (Table S2).

### 2.3. HCC Significant Targets

HCC significant targets were retrieved from OncoDB.HCC [29] (http://oncodb.hcc.ibms.sinica.edu.tw/) and Liverome [30] (http://liverome.kobic.re.kr/index.php). OncoDB.HCC effectively integrates three datasets from public references to provide multidimension view of current HCC studies, as the first comprehensive oncogenomic database for HCC. Significant genes experimentally validated were selected [23]. Liverome is a curated database of liver cancer-related gene signatures, in which the gene signatures were obtained mostly from published microarray, proteomic studies and thoroughly curated by experts. Genes of occurrence frequency > 7 among various gene signatures were elected in Liverome [23]. The duplicated genes were removed from two databases and the corresponding targets were retained (Table S3). The predicted targets of main active ingredients of SBH were mapped to these targets to get common targets, which were regarded as candidate targets of SBH (Table S4).

### 2.4. Protein-Protein Interaction Data

The associated proteins of candidate targets of SBH were acquired from String [31] (https://string-db.org, ver 10.5), with the organism limited to “Homo sapiens”. String integrates known and forecasted protein-protein interactions and evaluates PPI with confidence score ranges (low confidence: score < 0.4; medium: 0.4-0.7; high: > 0.7). PPIs with high confidence scores were kept for further study.

### 2.5. Gene Ontology and Pathway Analysis

The GO biological process and molecular function were dissected via BINGO plug-in of Cytoscape. During this procedure, the significance level was set to 0.01, and organism was selected as Homo sapiens. The pathway analysis was executed by means of Reactome FI plug-in of Cytoscape.

### 2.6. Network Construction

The interaction networks were established as follows: (1) network between active ingredients and candidate targets of SBH; (2) network between common targets and associated human proteins that directly or indirectly interacted with candidate targets of SBH; (3) network among active ingredients, common targets, and pathways. The network construction was performed via utilizing visualization software Cytoscape [32] (version 3.2.1).

## 3. Results and Discussion

### 3.1. Ingredient - Target Network Analysis

**Figure 2:**
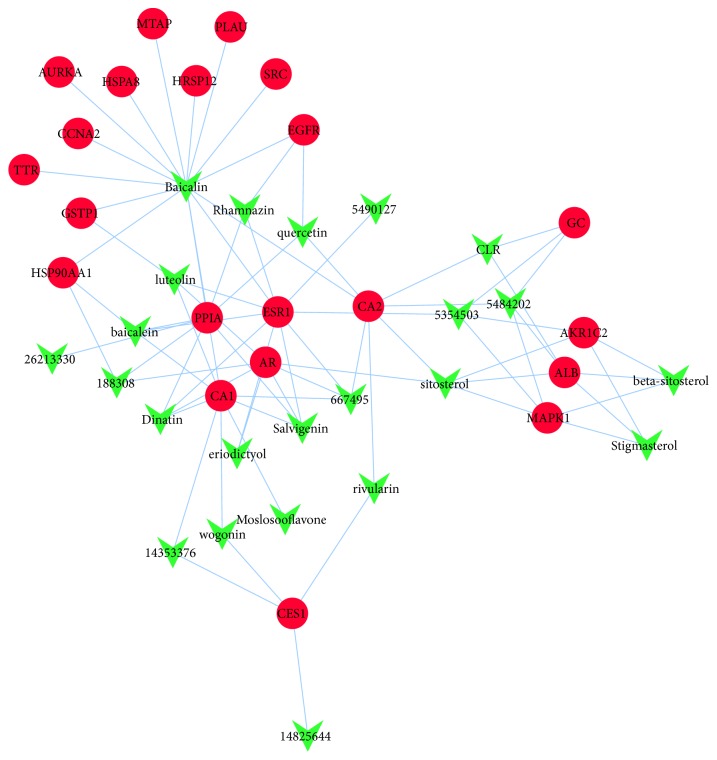
Ingredient-target network. Green arrows represent active ingredients in SBH. Red circles represent common targets between ingredient targets from SBH and HCC significant targets. Edges represent interaction between ingredients and targets.

As shown in Figure 2, the ingredient-target network was composed of 44 nodes (23 active ingredient nodes and 21 candidate target nodes) and 78 edges. In the network, a total of 23 active ingredients from Scutellariae Barbatae Herba were derived from TCMSP database, which was correspondent with the characteristic of multiple components for TCM. Most ingredient nodes were connected with multiple target nodes such as Baicalin, Dinatin, Sitosteryl acetate, etc., which coincided with the characteristic of multiple targets for TCM. We also found that many targets were hit by multiple ingredients. For example, ESR1 was modulated by ten ingredients containing Chrysin-5-methylether, Baicalin, Sitosteryl acetate, Dinatin, baicalein, Salvigenin, Rhamnazin, and so on. CES1 was regulated by multiple components covering 5-hydroxy-7,8-dimethoxy-2-(4-methoxyphenyl)chromone, 7-hydroxy-5,8-dimethoxy-2-phenyl-chromone, rivularin, and wogonin. The phenomenon implied that active ingredients of SBH might act on these targets synergistically. The network target nodes represented common targets from intersections between ingredient targets from SBH and HCC significant targets, and so Figure 2 not only displayed the relationship between active ingredients and ingredient targets but also reflected the connection for SBH resisting HCC.

PharmMapper has been widely applied for computational target identification and can provide the top 300 candidate targets for the query compound by default [33]. The targets with normalized fit score > 0.9 were employed as potential targets in this study. Several potential targets of active ingredients from SBH have been recognized in other studies. For example, baicalein was an indirect CAR activator and interfered with epidermal growth factor receptor (EGFR) signaling [34]. Wedelolactone, apigenin, and luteolin from Wedelia chinensis synergistically disrupted the AR, HER2/3, and AKT signaling networks and enhanced the therapeutic efficacy of androgen ablation in prostate cancer [35]. Luteolin was observed to decrease sorbitol accumulation in the rat lens under high-sorbitol conditions ex vivo via high inhibitory activity against AR and may be used as natural drugs for treating diabetic complications [36]. Luteolin was also identified as the small-molecule drug during identifying the differentially expressed genes including ESR1 in kidneys undergoing laparoscopic donor nephrectomy [37]. The protein expression of GSTP1 was mainly dominated by AhR pathway and luteolin inhibited the expression of drug-metabolizing enzymes by modulating Nrf2 and AhR pathways [38]. Quercetin downregulated the expression of EGFR and modulated this signal pathway on the liver-induced preneoplastic lesions in rats [39]. On the other hand, quercetin was considered an effective anti-cancer agent against breast cancer, human head and neck squamous carcinoma, prostate cancer, oral cancer, and pancreatic tumor [40–44]. The above literature data indicated the accuracy of target prediction with PharmMapper.

### 3.2. HCC Targets' PPI Network Analysis

**Figure 3:**
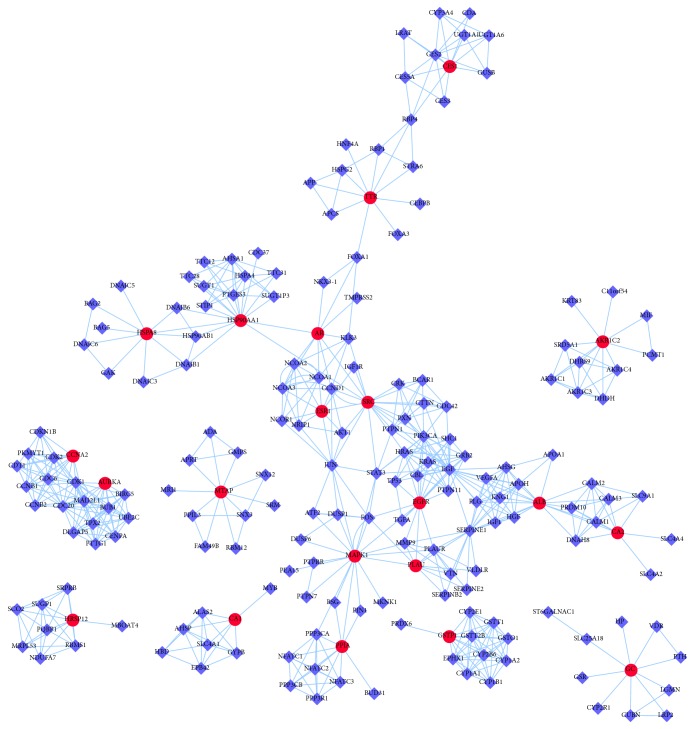
HCC targets' PPI network. Purple Diamonds represent associated human proteins that directly or indirectly interacted with common targets. Red circles represent common targets between ingredient targets from SBH and HCC significant targets.

The PPI network was composed of candidate targets of SBH and associated human proteins that directly or indirectly interacted with those in Figure 3. The network was composed of 200 nodes (21 candidate target nodes and 179 associated target nodes) and 622 edges. The network systematically and thoroughly summarized internal net of SBH response to HCC. The main section of the network covered 13 (61.9%) candidate target nodes and 107 (59.8%) associated target nodes, which might play the leading role in the process of pharmacological effects for SBH.

### 3.3. GO and Reactome Analysis

**Figure 4:**
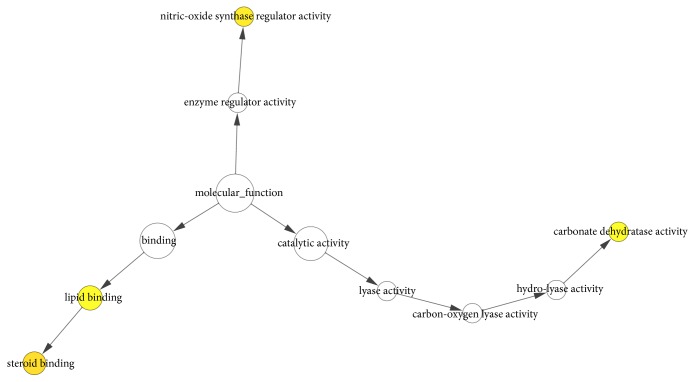
Gene Ontology (GO) Molecular Function Analysis for candidate targets of SBH. The yellow nodes indicate significant enrichment of molecular function terms. The larger the yellow node, the more the term enrichment. The darker the color, the smaller the P value (p < 0.01).

**Figure 5:**
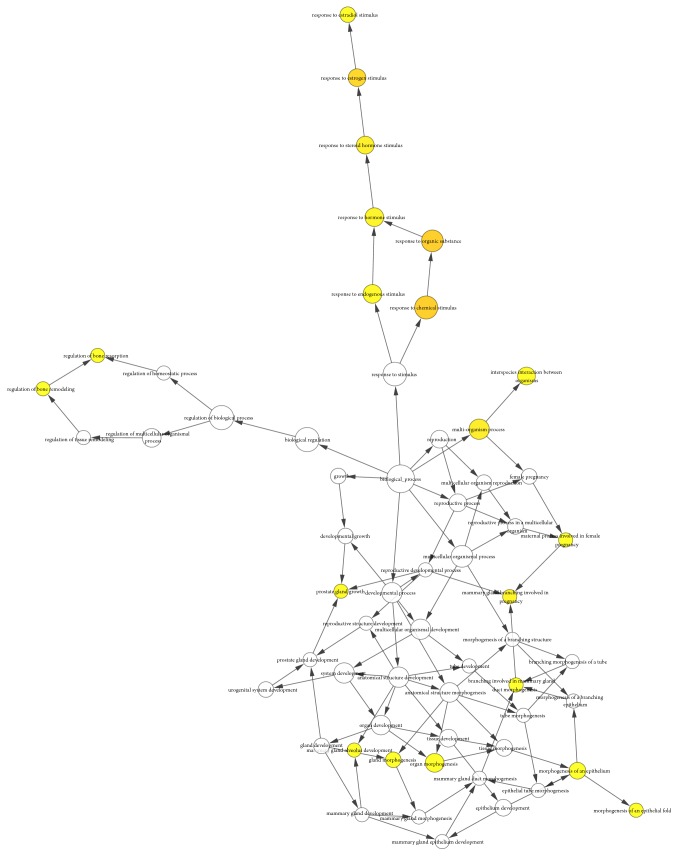
Gene Ontology (GO) Biological Process Analysis for candidate targets of SBH. The yellow nodes indicate significant enrichment of biological process terms. The larger the yellow node, the more the term enrichment. The darker the color, the smaller the P value (p < 0.01).

To further excavate the significance of common targets, the GO molecular function and biological process were analysed via BINGO plug-in of Cytoscape. As shown in Figure 4 and Table S5, SBH effected HCC by regulating four principal molecular functions, namely, steroid binding, nitric-oxide synthase regulator activity, carbonate dehydratase activity, and lipid binding. As shown in Figure 5 and Table S6, SBH mainly participated in 20 biological processes containing response to organic substance, response to chemical stimulus, response to estrogen stimulus, multi-organism process, response to steroid hormone stimulus, and so on. The yellow nodes indicated significant enrichment of GO terms. The larger the yellow node, the more the term enrichment. The darker the color, the smaller the P value.

**Figure 6:**
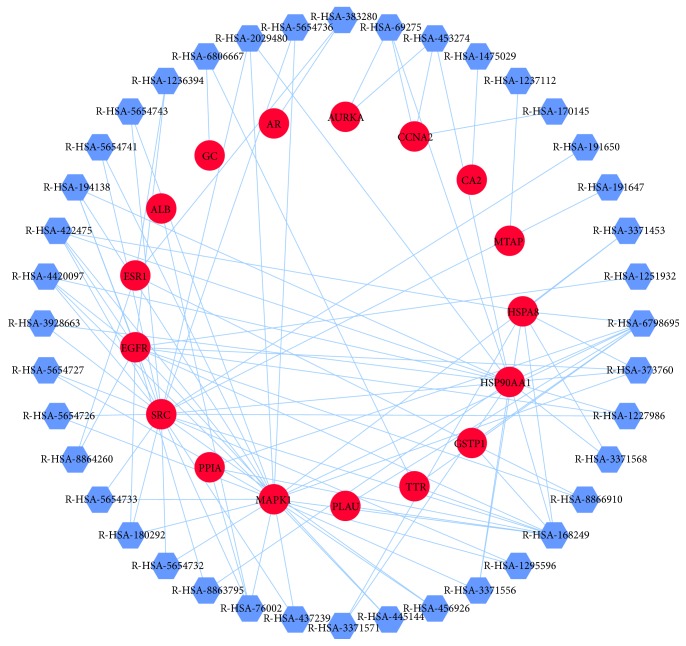
Target-pathway network. Blue hexagons represent enriched Reactome pathways. Red circles represent common targets between ingredient targets from SBH and HCC significant targets.

**Figure 7:**
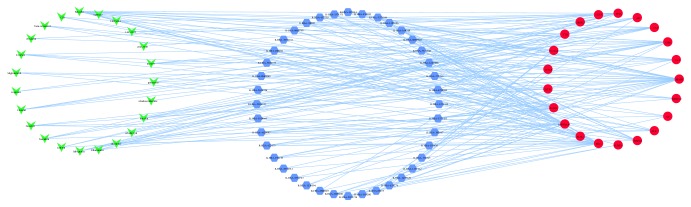
Ingredient-target-pathway network. Green arrows represent active ingredients in SBH. Red circles represent common targets between ingredient targets from SBH and HCC significant targets. Blue hexagons represent enriched Reactome pathways.

The pathway analysis was executed by means of Reactome FI plug-in of Cytoscape. As shown in Table S7, the 21 common targets were involved in 40 Reactome pathways ( p < 0.01). It was found that SBH fought against HCC mainly depending on neutrophil degranulation, L1CAM interactions, signaling by ERBB2, attenuation phase, TFAP2 (AP-2) family regulating transcription of growth factors and their receptors, innate immune system, etc. Then the relevant target-pathway network and ingredient-target-pathway network were constructed with nodes consistent with ingredients, targets, pathways, and edges indicating interactions, respectively, in Figures 6 and 7. The network also indicated that SBH possessed multiple components, multiple targets, and multiple pathways against HCC.

**Figure 8:**
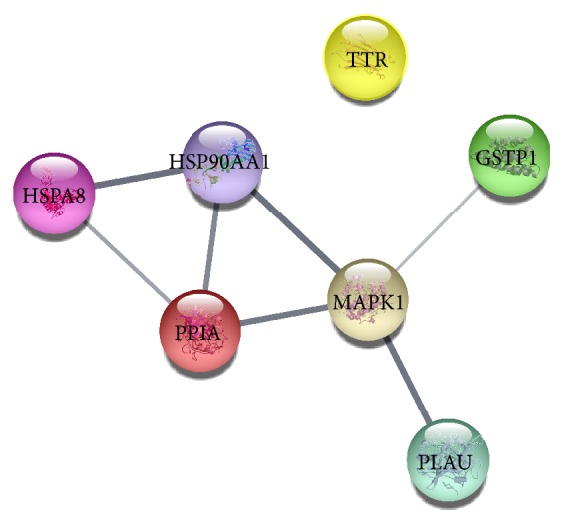
Interaction network of all hit genes.

**Figure 9:**
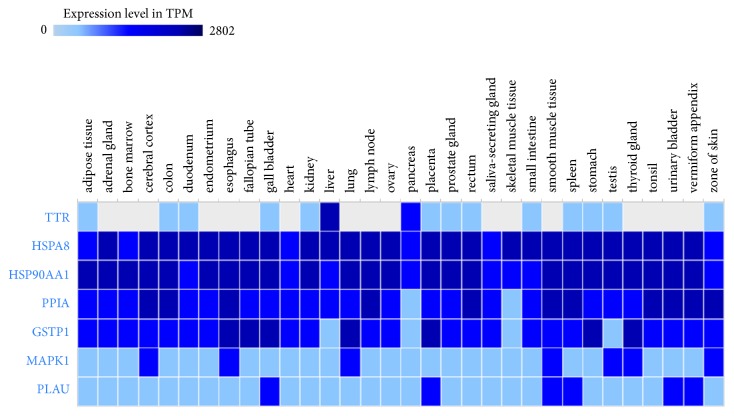
Hit genes analysed by Expression Atlas. X-axis represents 32 different tissues. Y-axis represents hit genes.

Previous studies have reported that L1CAM interactions, signaling by ERBB2, attenuation phase, TFAP2 (AP-2) family regulating transcription of growth factors and their receptors, and innate immune system played important roles in HCC [45–49], which was fully in support of the reliability of network analysis prediction. Performing in-depth analysis of the first-ranked signaling pathway, we found that neutrophil degranulation was involved in seven genes in this study, namely, HSPA8, HSP90AA1, GSTP1, TTR, PLAU, MAPK1, PPIA. The roles of these genes in HCC have also been reported in previous literature. High GSTP1 could inhibit cell proliferation by reducing AKT phosphorylation and provide a better prognosis in hepatocellular carcinoma [50]. The high-ranking gene MAPK1 was confirmed as an important target involved in hepatocarcinogenesis [51]. The VEGF/VEGFR2 pathway might be associated with HCC recurrence in patients expressing high levels of HSP90AA1/HSPA8 [52]. PPIA regulated cell growth and could serve as a novel marker and therapeutic molecular target for HCC patients [53]. Serum TTR might be useful for predicting the prognosis of HCC patients [54]. The String database was employed to construct an interaction network of all hit genes. Interestingly, HSPA8, HSP90AA1, GSTP1, PLAU, MAPK1, and PPIA formed a network of interactions and TTR was independent of the network in Figure 8. All hit genes were further analysed through Expression Atlas, which provided RNA-seq of coding RNA from tissue samples of 122 human individuals representing 32 different tissues. As shown in Figure 9 and Table S8, GSTP1, PLAU, and MAPK1 rendered lower expression, and HSPA8, HSP90AA1, and PPIA were expressed in various organizations without obvious differences. It was surprising that TTR was highly and specially expressed in the liver tissue. TTR might play a crucial role in SBH against HCC.

## 4. Conclusion

23 of 29 active herbal ingredients were determined by OB and DL from TCMSP database; their 3D molecular structures were obtained from PubChem database and respective targets were predicted via PharmMapper Database. HCC significant targets were retrieved from OncoDB.HCC and Liverome, which were mapped to predicted targets of active ingredients of SBH to get 21 common targets regarded as candidate targets of SBH. The 21 common targets were analysed by Cytoscape plug-ins. The results revealed that SBH effected HCC by regulating four principal molecular functions and 20 biological processes. The pathway analysis suggested the 21 common targets were involved in 40 Reactome pathways. The first-ranked signaling pathway and hit genes were further analysed through network related tools. We found that TTR was highly and specially expressed in the liver tissue. TTR might play a crucial role in neutrophil degranulation pathway during SBH against HCC. TTR might become a therapeutic target of HCC and further experiments are needed to provide support for our findings. This study provided a systematic view of anti-hepatoma mechanisms of Scutellariae Barbatae Herba from a network-based perspective.

## Data Availability

The data used to support the findings of this study are available from Supplementary Materials.

## Supplementary Materials

Supplementary Materials Table S1: 29 active ingredients from TCMSP. Table S2: ingredients in SBH and corresponding targets. Table S3: HCC targets. Table S4: candidate targets of SBH. Table S5: Gene Ontology (GO) Molecular Function Analysis for candidate targets of SBH. Table S6: Gene Ontology (GO) Biological Process Analysis for candidate targets of SBH. Table S7: Reactome analysis for candidate targets of SBH. Table S8: hit genes analysed by Expression Atlas.
